# Associations of the metabolic syndrome and its components with cognitive impairment in older adults

**DOI:** 10.1186/s12877-019-1073-7

**Published:** 2019-03-07

**Authors:** Insa Feinkohl, Jürgen Janke, Daniel Hadzidiakos, Arjen Slooter, Georg Winterer, Claudia Spies, Tobias Pischon

**Affiliations:** 1 Molecular Epidemiology Research Group, Max-Delbrueck-Center for Molecular Medicine in the Helmholtz Association (MDC), Berlin, Germany; 2 Charité – Universitaetsmedizin Berlin, corporate member of Freie Universitaet Berlin, Humboldt-Universitaet zu Berlin, and Berlin Institute of Health (BIH), Berlin, Germany; 3 University Medical Center Utrecht, Utrecht, the Netherlands; 4 MDC/BIH Biobank, Max-Delbrueck-Center for Molecular Medicine in the Helmholtz Association (MDC), and Berlin Institute of Health (BIH), Berlin, Germany

**Keywords:** Cognitive impairment, Epidemiology, High-density lipoprotein, Glucose, Metabolic syndrome, Triglycerides

## Abstract

**Background:**

The metabolic syndrome (MetS) is an established cardiovascular risk factor. Here, we investigated its role in cognitive impairment.

**Methods:**

Baseline data from 202 participants (aged 65 to 87 years) of the BioCog study were used. All were free of clinical dementia (MMSE≥24/30). Cognitive impairment was defined as the lowest tertile of a cognitive summary score. Multiple logistic regression analyses examined associations of body mass index (BMI), triglycerides (TG), high-density lipoprotein (HDL-C), glucose and glycated hemoglobin A1c (HbA1c) levels with the odds of cognitive impairment. MetS was defined as ≥3 of its 5 components obesity (BMI ≥ 30 kg/m^2^), elevated TG (TG ≥1.7 mmol/L), reduced HDL-C (males: < 1.0 mmol/L; females: < 1.3 mmol/L), elevated glucose (glucose ≥5.5 mmol/L and/or diagnosed diabetes) and elevated blood pressure (history of hypertension). Analyses controlled for age, sex and smoking history.

**Results:**

Lower HDL-C was significantly associated with a higher odds of cognitive impairment (OR 2.70 per 1 mmol/L reduction; 95% CI 1.25, 5.56; *p* = 0.011), whereas BMI, TG, glucose and HbA1c were not (all *p* > 0.05). Results for HDL-C were similar when HDL-C, glucose, BMI and TG were entered into a single model (OR 2.56 per 1 mmol/L reduction, 95% CI 1.09, 5.88, *p* = 0.031) and when cerebrovascular disease and coronary heart disease were additionally controlled for (OR 2.56 per 1 mmol/L reduction, 95% CI 1.06, 6.25, *p* = 0.036). Among the 5 MetS components, participants with elevated TG were at 2-fold increased odds of impairment (OR 2.09, 95% CI 1.08, 4.05, *p* = 0.028) including when the remaining 4 MetS components were entered (OR 2.23, 95% CI 1.07, 4.65, *p* = 0.033), but the finding was no longer statistically significant when cerebrovascular disease and coronary heart disease were additionally controlled for (*p* = 0.11). Presence of MetS and of obesity, reduced HDL-C, elevated glucose or elevated blood pressure were not significantly associated with impairment (all *p* > 0.05).

**Conclusion:**

Our findings support low HDL-C as an independent risk marker of cognitive impairment in older age. The need for research into mediatory and confounding factors, and re-evaluation of traditional cut-off points is highlighted.

**Trial registration:**

The study was registered on 15th October 2014 at clinicaltrials.gov (NCT02265263).

## Background

The metabolic syndrome (MetS) is a cluster of metabolic abnormalities, including abdominal obesity, elevated blood pressure (BP), elevated blood glucose levels, low high-density lipoprotein cholesterol (HDL-C) levels, and elevated triglyceride (TG) levels, and is suggested to play a major role in the development of cardiovascular disease (CVD) and type 2 diabetes mellitus [1]. Although its definition had been a matter of debate [1], MetS is now a widely accepted concept [2], and has been used across multiple populations to assess cardiovascular and mortality risk [3]. For example, it was estimated that 11 million deaths world-wide can be attributed to MetS annually [3]. Although MetS has traditionally primarily been linked to CVD, studies suggest that MetS [4–7] and metabolic abnormalities more generally [7–9] may also be related to cognitive impairment as a type of organ dysfunction that burdens the global economy to extents similar to CVD [10]. Chronically elevated blood glucose levels, for instance, have consistently been associated with an increased risk of future cognitive impairment [11].

MetS [12] and its contributing parameters of metabolic dysfunction (e.g., [13]) are hugely prevalent in Western societies and on a global scale, but all are modifiable. This implies a potential for strategic improvement of public health that warrants clarification. We therefore examined associations of MetS, of each of its 5 components and of associated continuous parameters of metabolic dysfunction with cognitive impairment in a community-based sample of older adults without clinical dementia.

## Method

### Study design

We analyzed cross-sectional associations of MetS with cognitive impairment in the Biomarker Development for Postoperative Cognitive Impairment in the Elderly (BioCog) study (http://www.biocog.eu). The primary aim of the study is to identify biomarkers predictive of post-operative cognitive impairment in patients undergoing elective surgery at study sites in Utrecht, the Netherlands, and Berlin, Germany. Details on recruitment procedures and study protocol have been reported elsewhere [14] and the study was registered on 15th October 2014 at clinicaltrials.gov (NCT02265263). In brief, patients were eligible to participate if they were aged ≥65 years, Caucasian, scheduled for elective surgery of any type with operative time ≥ 60 min and with an expected post-operative hospital treatment period of at least 7 days, and if they scored normal on a screening tool for dementia (Mini Mental State Examination, MMSE≥24/30). Of 7727 patients screened for inclusion, 933 were recruited between November 2014 and April 2017. Here, we report on baseline metabolic and cognitive data that were collected before surgery from the first 400 of those patients. Participants with missing data on any of the 5 MetS components or any missing cognitive data were excluded from our analysis.

### Clinical interview and physical examination

Participants self-reported on smoking history and sociodemographic parameters. Arterial hypertension, diabetes, a history of transient ischemic attacks (TIA), a history of stroke and coronary heart disease (CHD) were ascertained from a combination of self-report and local hospital records on pre-existing conditions and medication. Weight and height were measured to calculate body mass index (BMI).

### Biomarker measurement

Blood was collected immediately before induction of anesthesia in a supine position and following an overnight fast. HbA1c was measured in a laboratory adjacent to the respective hospital site. Blood was additionally centrifuged and serum samples stored at − 80 °C for shipment to a central biobank repository. Samples were later retrieved from that biobank for measurement of glucose, TG and HDL-C levels. Those analyses were performed at a single laboratory. Because samples stored at the biobank were insufficient for *N* = 16 participants of our analysis sample, data on glucose, TG and HDL-C were used from the immediate laboratory adjacent to the hospital site for those 16 participants. Sensitivity analyses revealed no influence of analysis laboratory on any of the results reported here (data not shown). For one participant, blood was collected after induction of anesthesia but before incision. Their data were not excluded.

### Definition of metabolic syndrome

In accordance with standardized criteria [15], we used a slightly modified definition of MetS (Table 1). BMI was used to define obesity instead of waist circumference, since waist circumference was not measured in our study.

**Table 1:** Definition of metabolic syndrome^a^

Component	Standard criteria^b^	Criteria used in present study
Elevated waist circumference	Population- and country-specific definitions	BMI ≥ 30 kg/m^2^
Elevated TG	TG ≥ 150 mg/dL (1.7 mmol/L), or drug treatment	Fasting TG ≥150 mg/dl (1.7 mmol/L)
Reduced HDL-C	HDL-C < 40 mg/dL (1.0 mmol/L) in males; HDL-C < 50 mg/dl (1.3 mmol/L) in females; or drug treatment	HDL-C < 40 mg/dl (1.0 mmol/L) in males HDL-C < 50 mg/dl (1.3 mmol/L) in females
Elevated blood pressure	Systolic ≥130 and/or diastolic ≥85 mmHg, or drug treatment	Hypertension based on self-report and/or local hospital records
Elevated glucose	≥100 mg/dL in plasma, or drug treatment	1. Fasting blood glucose^c^ ≥100 mg/dL (5.5 mmol/L) (if not fasted, HbA1c ≥42 mmol/mol^d^) and/or 2. Diabetes based on self-report and/or local hospital records

^a^The metabolic syndrome is defined as the presence of at least 3 of the 5 components

^b^Consensus statement [15]

^c^Glucose measured in serum (nearly identical to plasma; [59])

^d^In the present sample, all participants were fasted

### Cognitive examination

Participants underwent neuropsychological testing in a quiet hospital room usually on the day before surgery. The MMSE was used to screen for clinical dementia for inclusion into the study, before a series of computer-based (Cambridge Neuropsychological Test Automated Battery, CANTAB®; Cambridge Cognition Ltd.) and paper-pencil tests were performed: Paired Associates Learning (PAL), Verbal Recognition Memory (VRM), Spatial Span (SSP), Simple Reaction Time (SRT), Trail-Making Test-B (TMT-B), and Grooved Pegboard (GP). Principal component analysis (PCA) with extraction of factors with Eigenvalue > 1 was applied to the 6 cognitive tests to derive a score of global cognitive ability (‘*g*’) [16]. *G* is unaffected by test-specific measurement error, produces more reliable results compared with individual cognitive tests and typically accounts for around 40% of variance [17]. *G* is independent of cognitive test battery [18], and all aforementioned tests have been used to calculate *g* in the past (e.g., [18]). Visual inspection of a scree plot confirmed presence of a single factor (Eigenvalue 2.37) explaining 39.53% of variance in the data. Standardized residuals of that factor were saved to obtain an operant latent variable *g* (factor loadings TMT-B, 0.77; PAL, 0.71; GP, 0.67; VRM, 0.56; SSP, 0.54; SRT, 0.49). ‘Cognitive impairment’ was defined as scoring in the lowest tertile of *g* and was the outcome of interest in our analysis. As a screening tool for dementia [19], the MMSE was not used to calculate *g*.

### Statistical analysis

Data for MMSE, TMT-B, SRT and GP were log transformed prior to analysis to approximate normal distribution. An initial univariate analysis of variance (ANOVA) compared MMSE scores across tertiles of *g*; a chi^2^ test compared associations of MMSE< 27 (indicative of prodromal dementia) with presence of cognitive impairment.

Participants were divided into quartiles based on the respective distributions of HbA1c, glucose, TG, HDL-C and BMI. We then used multiple logistic regression to examine the association of each with odds of cognitive impairment using the lowest quartile as the reference category. In addition, we used each on a continuous scale. For each analysis, we ran three regression models: Model 1 was adjusted for age, sex and smoking. Model 2 included age, sex, smoking, BMI, TG, HDL-C, and either glucose or HbA1c. Model 3 additionally included CHD, TIA and stroke. To check for non-linearity in the association with cognitive impairment, we subsequently added quadratic terms into the respective final model (Model 3).

We next categorized MetS and each of its components based on established definitions (Table 1) and studied their association with odds of cognitive impairment using multiple logistic regression. Again, Model 1 was adjusted for age, sex and smoking, Model 2 included age, sex, smoking and all 5 MetS components, and Model 3 additionally controlled for CHD, TIA and stroke.

We then studied the association of the number of abnormal MetS components with the odds of cognitive impairment using 0 abnormal components as reference category. For the purpose of this analysis, the groups with 4 or 5 components were merged due to small participant numbers in these groups. Finally, the number of MetS components (range 0 to 5) was entered into a multiple logistic regression model. For these analyses, Model 1 controlled for age, sex and smoking, and Model 2 additionally controlled for CHD, TIA and stroke. All results remained unchanged following exclusion of 2 underweight participants (BMI ≤ 18.5 kg/m^2^) and 1 participant with very high TG (28.9 mmol/L) in a separate analysis unless stated otherwise. SPSS version 18.0 (IBM Corporation, New York) was used.

## Results

### Sample characteristics

A total of 202 participants enrolled into the study had complete cognitive and MetS data. Demographic, metabolic and cognitive characterization of the analysis sample is shown in Table 2. Participants were most commonly scheduled for orthopedic, gynecologic/urologic or general surgery and a majority had elevated BP (60.9%) and elevated fasting glucose (61.9%) respectively (Table 1). Obesity was present in 22.3%, TG were elevated in 29.7%, and HDL-C was reduced in 34.2% of participants. Seventy-two participants (35.6%) fulfilled the criteria for MetS.

**Table 2:** Demographic, metabolic and cognitive sample characteristics

	Means ± SD, median (interquartile range) or n of total *N* = 202 analysis sample	% of N
Study site		
UMC Utrecht, n (%)	33	16.3%
Charité Berlin Campus Virchow, n (%)	114	56.4%
Charité Berlin Campus Mitte, n (%)	55	27.2%
Male, n (%)	121	59.9%
Age, years, mean ± SD	72.12 ± 4.74	
Smoking history, n (%)		
Missing	28	13.9%
Never smokers	54	26.7%
Former smokers	93	46.0%
Current smokers	27	13.4%
History of coronary heart disease, n (%)	28	13.9%
History of stroke, n (%)	9	4.5%
History of transient ischemic attack, n (%)	6	3.0%
History of diabetes, n (%)	39	19.3%
Non-insulin dependent diabetes, n (%)	23	11.4%
Insulin-dependent diabetes, n (%)	16	7.9%
History of dyslipidemia, n (%)	40	19.8%
Body mass index (BMI; kg/m^2^), mean ± SD	27.12 ± 4.39	
Serum glucose (mmol/L), median (interquartile range)	5.77 (5.27–6.49)	
HbA1c^a^ (mmol/mol), mean ± SD	39.68 ± 8.20	
Triglycerides (TG) (mmol/L), median (interquartile range)	1.31 (1.04–1.79)	
Total cholesterol^a^ (mmol/L), mean ± SD	4.88 ± 1.12	
Low-density lipoprotein^a^ (LDL-C) (mmol/L), mean ± SD	3.11 ± 0.98	
High-density lipoprotein (HDL-C) (mmol/L), mean ± SD	1.27 ± 0.43	
BMI categories, n (%)		
Underweight (BMI ≤ 18.5 kg/m^2^), n (%)	2	1.0%
Normal/overweight (BMI 18.6–29.9 kg/m^2^), n (%)	155	76.7%
Obesity (BMI ≥30 kg/m^2^), n (%)	45	22.3%
Elevated blood pressure^b^, n (%)	123	60.9%
Elevated fasting glucose^b^, n (%)	125	61.9%
Elevated TG^b^, n (%)	60	29.7%
Reduced HDL-C^b^, n (%)	69	34.2%
Metabolic syndrome (MetS)^b^, n (%)	72	35.6%
Number of MetS components^b^		
0	27	13.4%
1	41	20.3%
2	62	30.7%
3	41	20.3%
4	21	10.4%
5	10	5.0%
Factor of global ability *g*, mean ± SD	−0.06 ± 1.01	
Mini Mental State Examination (MMSE)^a^, median (interquartile range)	29 (28–30)	
MMSE< 27^a^, n (%)	12	6.0%

^a^for HbA1c, *N* = 155; for total cholesterol, *N* = 158; for LDL-C, *N* = 157; for MMSE, *N* = 200

^b^for definition, see Table 1

### Comparison of Mini-Mental State Examination (MMSE) scores across tertiles of *g*

Scores on the MMSE differed statistically significantly across tertiles of *g* (F (2, 197) = 12.38; *p* < 0.001; ŋp^2^ = 0.11). Participants with cognitive impairment (those scoring in the lowest tertile *g*) had lower MMSE (geometric mean 28.1, 95% CI 27.8, 28.4) relative to the second (geometric mean 28.7, 95% CI 28.4, 29.0) and third tertiles (geometric mean 29.1, 95% CI 28.8, 29.4) (pairwise comparison range *p* < 0.001 to *p* = 0.110). Of 12 participants with MMSE< 27, 8 had cognitive impairment when defined from *g* (chi^2^ (1, *N* = 200) = 5.42; *p* = 0.020).

### Age- and sex associations with cognitive impairment

Age was directly associated with cognitive impairment. Each 5-year increase in age was associated with a 1.79-fold increased odds of impairment (OR 1.79 per 5-year increment, 95% CI 1.30, 2.47; *p* < 0.001). Sex was unrelated to impairment in the same model (male versus female, OR 0.70, 95% CI 0.38, 1.28; *p* = 0.25).

### Continuous metabolic parameters and odds of cognitive impairment

The odds of cognitive impairment according to each of the continuous metabolic parameters and their quartiles are shown in Table 3. HDL-C quartiles were significantly associated with cognitive impairment (*p*_trend_ across quartiles adjusted for age, sex, smoking = 0.004). Thus, persons in the highest versus lowest quartile of HDL-C had a 0.28-fold odds (95% CI 0.11–0.71). The association also survived addition of BMI, TG, glucose, CHD, TIA and stroke into the model (*p*_trend_ across quartiles = 0.023). On a continuous scale, in the fully adjusted model, each 1 unit mmol/L higher HDL-C concentration was associated with a 0.39-fold odds (OR 0.39; 95% CI 0.16, 0.94; *p* = 0.036) of cognitive impairment.

**Table 3:** Odds of cognitive impairment according to continuous metabolic parameters

	Quartiles				*p*_trend_	Continuous parameters	
	1	2	3	4		OR (95% CI) per unit increment	*p*-value
Body mass index							
Cut-point (kg/m^2^)	≤24.15	24.16–26.70	26.71–29.35	≥29.36			
n with cognitive impairment / N total	19 / 51	18 / 51	18 / 50	17 / 50			
Model 1 OR (95% CI)	1.00 (Reference)	0.96 (0.41, 2.25)	1.17 (0.50, 2.75)	1.02 (0.43, 2.41)	0.971	0.99 (0.92, 1.06)	0.772
Model 2 OR (95% CI)	1.00 (Reference)	1.06 (0.41, 2.70)	1.11 (0.43, 2.87)	0.64 (0.23, 1.80)	0.707	0.95 (0.88, 1.03)	0.205
Model 3 OR (95% CI)	1.00 (Reference)	0.98 (0.37, 2.64)	1.18 (0.44, 3.13)	0.59 (0.20, 1.75)	0.602	0.95 (0.88, 1.03)	0.238
Triglycerides							
Cut-point (mmol/L)	≤1.04	1.05–1.31	1.32–1.79	≥1.80			
n with cognitive impairment / N total	17 / 53	14 / 49	23 / 50	18 / 50			
Model 1 OR (95% CI)^a^	1.00 (Reference)	0.92 (0.38, 2.24)	2.20 (0.94, 5.19)	1.58 (0.66, 3.77)	0.167	1.11 (0.93, 1.32)	0.241
Model 2 OR (95% CI)^a^	1.00 (Reference)	1.07 (0.39, 2.91)	2.22 (0.82, 5.98)	0.91 (0.31, 2.73)	0.259	1.02 (0.88, 1.18)	0.835
Model 3 OR (95% CI)^a^	1.00 (Reference)	1.08 (0.38, 3.07)	2.08 (0.75, 5.76)	0.73 (0.23, 2.31)	0.237	1.02 (0.88, 1.18)	0.791
High-density lipoprotein cholesterol							
Cut-point (mmol/L)	≤1.01	1.02–1.27	1.28–1.55	≥1.56			
n with cognitive impairment / N total	28 / 54	10 / 52	21 / 52	13 / 44			
Model 1 OR (95% CI)	1.00 (Reference)	0.23 (0.09, 0.57)	0.65 (0.29, 1.48)	0.28 (0.11, 0.71)	0.004	0.37 (0.18, 0.80)	0.011
Model 2 OR (95% CI)	1.00 (Reference)	0.25 (0.09, 0.65)	0.57 (0.22, 1.44)	0.26 (0.09, 0.80)	0.017	0.39 (0.17, 0.92)	0.031
Model 3 OR (95% CI)	1.00 (Reference)	0.28 (0.10, 0.75)	0.53 (0.20, 1.41)	0.22 (0.07, 0.70)	0.023	0.39 (0.16, 0.94)	0.036
Glucose							
Cut-point (mmol/L)	≤5.27	5.28–5.77	5.78–6.49	≥6.50			
n with cognitive impairment / N total	21 / 54	14 / 48	14 / 52	23 / 48			
Model 1 OR (95% CI)	1.00 (Reference)	0.56 (0.23, 1.35)	0.56 (0.24, 1.33)	1.62 (0.71, 3.70)	0.053	1.19 (0.99, 1.43)	0.068
Model 2 OR (95% CI)	1.00 (Reference)	0.66 (0.26, 1.66)	0.42 (0.16, 1.10)	1.58 (0.61, 4.08)	0.062	1.19 (0.97, 1.46)	0.094
Model 3 OR (95% CI)	1.00 (Reference)	0.62 (0.23, 1.66)	0.45 (0.16, 1.21)	1.84 (0.69, 4.91)	0.045	1.21 (0.97, 1.51)	0.086
HbA1c							
Cut-point (mmol/mol)	≤35.5	35.6–38.8	38.9–42.1	≥42.2			
n with cognitive impairment / N total	15 / 46	14 / 37	12 / 38	17 / 34			
Model 1 OR (95% CI)	1.00 (Reference)	1.26 (0.49, 3.27)	0.65 (0.24, 1.75)	2.15 (0.83, 5.54)	0.142	1.03 (0.99, 1.08)	0.137
Model 2 OR (95% CI)	1.00 (Reference)	1.24 (0.44, 3.46)	0.54 (0.18, 1.63)	1.71 (0.61, 4.80)	0.235	1.03 (0.99, 1.08)^b^	0.137
Model 3 OR (95% CI)	1.00 (Reference)	0.75 (0.24, 2.39)	0.55 (0.18, 1.73)	1.47 (0.49, 4.36)	0.420	1.04 (0.99, 1.09)^b^	0.115

Results shown for logistic regression analyses with outcome cognitive impairment. *p*-value for trend (2-sided) based on the respective median within quartiles, used as a continuous variable, and analyzed using the Wald chi^2^ statistic. CI, confidence interval; OR, odds ratio

^a^results largely unchanged following exclusion of *N* = 1 outlier with high TG levels (28.9 mmol/L)

^b^in these models, HDL-C was significantly associated with cognitive impairment (Model 2: OR 0.27, 95% CI 0.09, 0.79, *p* = 0.016; Model 3: OR 0.28, 95% CI 0.09, 0.83, *p* = 0.022; for TG and BMI, all *p* > 0.05 in these models)

Model 1: adjusted for age, sex, smoking

Model 2: Model 1 + TG quartiles, HDL-C quartiles, BMI quartiles, glucose quartiles (for quartile analyses) or Model 1 + TG, HDL-C, BMI and glucose (for continuous parameters) (analysis *N* = 202), or for HbA1c: Model 1 + TG quartiles, HDL-C quartiles, BMI quartiles (for HbA1c quartile analyses) or Model 1 + TG, HDL-C and BMI (for analysis of HbA1c as continuous parameter) (analysis *N* = 155)

Model 3: Model 2 + CHD, TIA, stroke

Higher glucose levels were also related to a higher odds of cognitive impairment in the fully adjusted model (*p*_trend_ across quartiles = 0.045). On a continuous scale, 1 mmol/L higher glucose levels were associated with a statistically non-significant trend for a 1.21-fold odds (95%-CI 0.97–1.51; *p* = 0.086) of cognitive impairment. BMI, TG levels, and HbA1c concentrations were not substantially related to cognitive impairment in these analyses. To test for non-linearity we added quadratic terms of the metabolic parameters to each of the fully adjusted models; however, none of these quadratic terms were statistically significant (HDL-C, *p* = 0.407; TG, *p* = 0.556; BMI, *p* = 0.788; glucose, *p* = 0.282; HbA1c, *p* = 0.849), suggesting that non-linear models did not improve model fit.

### Metabolic syndrome, the 5 MetS components and odds of cognitive impairment

Participants with elevated TG were at 2.09-fold odds of cognitive impairment in analyses controlling for age, sex and smoking (OR 2.09, 95% CI 1.08, 4.05, *p* = 0.028) and when obesity, reduced HDL-C, elevated glucose and elevated BP were additionally adjusted for (OR 2.23, 95% CI 1.07, 4.65, *p* = 0.033; Table 4). Addition of CHD, TIA and stroke into the model led to statistically non-significant results, however (OR 1.86; 95% CI 0.87, 4.00; *p* = 0.110). Obesity, reduced HDL-C, elevated glucose and elevated BP were each not associated with cognitive impairment (all *p* > 0.05; see Table 4). The presence of MetS was not significantly related to cognitive impairment (OR adjusted for age, sex, smoking 1.38; 95% CI 0.74, 2.60; *p* = 0.310; Table 4). The number of MetS components was also not significantly associated with impairment (OR per number of component increment, adjusted for age, sex, smoking, 1.16, 95% CI 0.92, 1.45; *p* = 0.212; Table 5). Pairwise comparison showed a lower odds of cognitive impairment in the group with 1 MetS component compared with the reference group with 0 components in the fully adjusted model (OR 0.29; 95% CI 0.09, 0.95; *p* = 0.041) though no significant differences in the odds of cognitive impairment in participants with 2, 3 or 4/5 MetS components compared with the reference group were found (all *p* > 0.05; Table 5).

**Table 4:** MetS, each of the 5 MetS components and odds of cognitive impairment

	Model 1		Model 2		Model 3	
	OR (95% CI)	*p*	OR (95% CI)	*p*	OR (95% CI)	*p*
Metabolic syndrome	1.38 (0.74, 2.60)	0.310	–	–	1.25 (0.65, 2.42)	0.503
Obesity	1.07 (0.52, 2.23)	0.852	1.00 (0.46, 2.17)	0.997	1.08 (0.48, 2.43)	0.845
Elevated triglycerides	2.09 (1.08, 4.05)	0.028	2.23 (1.07, 4.65)	0.033	1.86 (0.87, 4.00)	0.110
Reduced high-density lipoprotein	1.19 (0.63, 2.23)	0.600	0.86 (0.42, 1.77)	0.691	0.87 (0.41, 1.82)	0.704
Elevated blood pressure	1.11 (0.60, 2.07)	0.740	1.04 (0.54, 2.00)	0.911	0.86 (0.44, 1.71)	0.668
Elevated glucose	1.12 (0.60, 2.08)	0.721	0.98 (0.51, 1.88)	0.948	1.09 (0.55, 2.14)	0.811

Results shown for logistic regression analyses for odds of cognitive impairment. CI, confidence interval; OR, odds ratio. For definitions of metabolic syndrome components, see Table 1. Model 1: separate models associated each exposure variable with cognitive impairment with adjustment for age, sex and smoking (*N* = 202). Model 2: single model including age, sex, smoking, obesity, elevated TG, reduced HDL-C, elevated blood pressure, elevated glucose (*N* = 202). Model 3: single model including age, sex, smoking, obesity, elevated TG, reduced HDL-C, elevated blood pressure, elevated glucose, CHD, TIA, stroke (*N* = 200). Model 3 is a separate model for MetS. Results largely unchanged following exclusion of *N* = 1 outlier with high TG levels (28.9 mmol/L)

**Table 5:** Number of MetS components and odds of cognitive impairment

Number of components	Model 1		Model 2	
	OR (95% CI)	*p*	OR (95% CI)	*p*
0	1.00 (Reference)^a^	–	1.00 (Reference)^b^	–
1	0.36 (0.11, 1.15)^a^	0.084	0.29 (0.09, 0.95)^b^	0.041
2	1.02 (0.38, 2.77)^a^	0.965	0.93 (0.34, 2.52)^b^	0.878
3	0.94 (0.32, 2.77)^a^	0.902	0.76 (0.26, 2.28)^b^	0.629
4/5^c^	1.23 (0.40, 3.77)^a^	0.713	0.99 (0.31, 3.12)^b^	0.982
Number of components (continuous)^d^	1.16 (0.92, 1.45)	0.212	1.11 (0.88, 1.41)	0.387

Model 1: adjusted for age, sex and smoking (*N* = 202). Model 2: adjusted for age, sex, smoking, CHD, TIA, stroke (*N* = 200)

^a^single model; ^b^single model

^c^due to small N in each, groups with 4 or 5 components were merged in this analysis

^d^range 0 to 5

## Discussion

In this cross-sectional analysis of a sample of older surgical patients without clinical dementia, participants with lower HDL cholesterol (HDL-C) and those with elevated triglycerides (TG) were at increased likelihood of being cognitively impaired. Individuals with higher glucose levels also had a higher odds of cognitive impairment, although these results became apparent only in quartile analyses. Importantly, the associations for HDL-C and glucose, but not for elevated TG, were largely independent of one another, of other parameters of metabolic dysfunction, and of age, sex, smoking and a history of macrovascular disease. Obesity and elevated blood pressure were not substantially associated with cognitive impairment.

Associations of mid-life obesity [8], mid-life dyslipidemia [20, 21] and mid-life hypertension [22] with later cognitive impairment including increased risk of Alzheimer’s disease and presence of Alzheimer’s-type neuropathology [23, 24] are well-established. In later life, these risk factors are more difficult to evaluate partly due to an influence of frailty [25], and previous studies of dyslipidemia in older age and cognitive impairment have produced mixed results. Null findings for diagnosed dyslipidemia [26] and for levels of total cholesterol [9], HDL-C [27] and TG [27, 28] are contrasted with studies showing an increased risk of cognitive impairment in people with low HDL-C [29, 30] or elevated TG in later life [31, 32]. Here, our data suggest a contribution of low HDL-C to cognitive impairment that could indicate a causal relationship. Indeed, HDL-C has vasoprotective and anti-inflammatory properties [33] so that reduced inflammation could be a plausible mediator of the association in our sample. TG levels correlate with atherogenic and pro-inflammatory triglyceride-rich lipoproteins (TRL) [34] which may directly promote cognitive impairment. Our findings also suggest a contribution of macrovascular disease to the association of elevated TG with cognitive impairment. Cerebrovascular disease could be a mediator in the relationship, for instance. We are unable to determine this from the present study. Nonetheless, irrespective of the issue of causality and mediatory processes, elevated TG and HDL-C both appear to be useful risk markers with potential for utility in clinical settings and could contribute to screening tool development.

The disparate findings on HDL-C as a continuous metabolic parameter versus the dichotomized MetS component ‘reduced HDL-C’ suggest that the latter at-risk group may not necessarily be well-captured by the standardized, sex-specific cut-off points that are currently in use [15]. Their reevaluation and update, including determination whether sex-specific cut-offs are necessary, may be warranted. We found no significant association when we used TG as a continuous variable or as quartiles in our analysis. In contrast, when based on the standardized cut-off point [15], elevated TG were significantly associated with cognitive impairment at least in largely unadjusted analyses, suggesting that this threshold is appropriate for cognitive risk prediction. Nevertheless, given the relatively small sample size, the results of our analysis need to be interpreted cautiously and require replication in larger samples.

Previous epidemiological research has consistently implicated hyperglycemia as detrimental to cognition. Irrespective of whether measured at midlife or later life, diabetes, pre-diabetes [35–37], and poorer glycemic control in people with diabetes [37] have been linked to increased risk of vascular-type impairment as well as Alzheimer’s disease [38]. Neurotoxic effects of glucose on the brain [39] and hyperglycemia-induced vascular damage [40] which appear to generate vascular impairment as well as facilitate neurodegeneration characteristic of Alzheimer’s disease [41] have been suggested as underlying the relationship. In our sample, we found evidence for a more complex role of glucose in cognitive impairment that became apparent only in quartile analyses and was not supported by analyses of HbA1c as an index of long-term glycemic control. The marginally significant result could thus reflect Type I error. The fact that – for consistency with standard definitions of MetS [15] – diagnosis of diabetes qualified for inclusion in the ‘elevated glucose’ group, may also have ‘diluted’ that group leading to non-significant results. Alternatively, the standardized cut-off point for ‘elevated glucose’ [15] may not be appropriate for our sample of surgical patients who may have had extended periods of fasting prior to blood collection or for whom fasting status may not have been recorded with sufficient rigor. The high prevalence of ‘elevated glucose’ (61.9%, albeit as aforementioned this included participants with diabetes) supports the latter possibility. The precise role of glucose in cognitive impairment thus remains to be explored further.

The evidence for obesity in older age as a risk factor for cognitive impairment is limited [8] with occasional implication of overweight, obesity and elevated waist circumference as protective factors [29, 42, 43]. Here, obesity and BMI both were not related to cognition. At the lower end of the body weight spectrum, the relationship may be affected by frailty [25] but results on obesity and BMI did not change when underweight participants were excluded from our analysis or when quadratic terms were added into the model. Elevated blood pressure, too, was unrelated to cognitive impairment contrasting with some other cross-sectional and longitudinal studies of older adults [22].

Previous studies of the MetS construct and cognitive impairment have occasionally produced null results similar to our own [44, 45]. However, others did report associations with impairment [7, 29, 30, 46, 47]. For instance, in the Singapore Longitudinal Ageing Study, participants with MetS were at 1.46-fold increased risk of mild cognitive impairment (MCI) during 6-year follow-up [7]. In the French Three-City Study of more than 7000 older adults, MetS – in line with its status as a vascular risk factor – was selectively associated with a 2.42-fold increased risk of impairment of vascular origin [29]. Finally, women with MetS were at 2.47-fold increased risk of poor memory 12 years later in a Finnish investigation [30] and a pooled analysis of three studies reported that MetS was overall associated with 2.95-fold increased risk of progression from MCI to dementia [48]. The Finnish study [30] and some others [49] additionally reported a linear relationship of the number of MetS components with cognitive risk, but we and others [29, 47] found no such evidence. Disparity of our results from previous studies could stem from our slightly modified definition of MetS, the cross-sectional study design, the surgical nature of our sample, and the high prevalence of MetS (35.6%) compared with those studies (12.9% [30]; 15.8% [29]; 22.4% [7]) but is in line with a recent systematic review of 25 studies which concluded that the evidence on associations of MetS with cognitive impairment in older age is insufficient at present [49]. A recent report of accumulation of beta amyloid in the brains of people with MetS [50] demonstrate the need for further research into the cognitive and neuropathological consequences of the syndrome.

Each MetS component (except obesity) can be modified through pharmaceutical treatment and the potential benefit of concurrent tackling of several components is being increasingly recognized. Thus, the ACCORD-MIND trial recently tested the effect of antidiabetic, lipid lowering and blood pressure lowering therapy, in a double 2 × 2 factorial design; however, neither improved glycemic control [51], nor improved lipid levels or blood pressure [52] affected the rate of cognitive decline during 40-month follow-up, suggesting that the epidemiological evidence linking elevated glucose, dyslipidemia and elevated blood pressure to cognitive impairment may be confounded. Further similarly complex trials are needed for clarification of the effects of strategic targeting of different metabolic parameters, as well as benefits of concurrent treatment, on cognitive risk.

Strengths of our study include a multi-center design and the use of a comprehensive cognitive test battery that was validated through comparison with an instrument commonly used to assess cognitive status. Consideration of several metabolic parameters in a single analysis was able to evaluate relative independence of each from one another in their relationship with cognition. Thus far the 5 MetS components have mainly been investigated in isolation. Only a few studies directly compared the components in terms of their association with cognitive risk and had implicated low HDL-C [30], elevated TG [29], hypertension [46] and, most frequently, hyperglycemia [26, 44] as independent risk markers. However, some limitations need to be considered. Surgical patients are at risk of developing post-operative cognitive impairment [53] and so are of special interest in terms of their cognitive status. To our knowledge the present study is the first to assess MetS and cognitive impairment in this type of sample. At the same time, the focus on surgical patients as well as self-selection bias preventing unwell patients to enroll limits the generalizability of our findings to the general population that includes healthy, community-dwelling individuals. Further, we used BMI as a proxy for central obesity [12] though strictly speaking central obesity can only be determined through direct measurement. We also did not consider MetS-related complications such as retinopathy in our analysis. The possibility of confounding of our statistically significant findings by unmeasured factors such as diet or physical activity, too, remains. Because ‘cognitive impairment’ was defined from a cognitive summary score, our results are not necessarily comparable to studies that used standardized constructs such as MCI. Due to the cross-sectional study design we were unable to evaluate participants’ metabolic function during the decades prior to enrolment and did not consider anti-hyperglycemic, anti-hypertensive and lipid-lowering treatment in our analysis. Associations of elevated blood pressure with cognitive impairment may thus have become apparent had we controlled for or stratified by treatment. We deem confounding of our findings on HDL-C by anti-dyslipidemia drugs unlikely given the balance of epidemiological and trial evidence which suggests a limited role of drugs such as statins or fibrates in cognitive decline [54–56]. In any event, the fact that we observed associations of HDL-C with cognitive impairment despite lacking data on treatment indicates that the underlying processes may be mechanistic and dose-dependent on lipid concentrations irrespective of whether they are treated. Finally, our sample was relatively small and so the fact that we did not find significant associations for some of the MetS components does not rule out that studies with larger sample size may be able to detect smaller effects. Further prospective, epidemiological studies comparing the contributions of each of the 5 components to cognitive risk are needed and should take advantage of a range of different types of samples to gain a full understanding of any sample-specific relationships of MetS with cognitive impairment. Researchers should additionally consider analysis of inflammatory markers, which may interact with MetS in determining cognitive outcome [57], as well as pre-morbid cognitive ability (which affects both cognitive ability and metabolic risk in older age [58]) to explore mediation and confounding.

In conclusion, in this cross-sectional analysis of older adults who were all free of clinical dementia and scheduled to undergo surgery, lower HDL-C and elevated TG were each associated with presence of cognitive impairment defined as reduced cognitive performance relative to the total sample. For HDL-C, but not for elevated TG, the finding was independent of age, sex, smoking, the remaining parameters of metabolic dysfunction, as well as of macrovascular disease. This suggests potential for a causal relationship. The MetS construct per se was not associated with cognition. Prospective studies should compare the cognitive risk associated with different parameters of metabolic dysfunction in view to identify at-risk individuals and to shed light on underlying pathophysiological mechanisms considering that the evidence for metabolic parameters as effective targets for intervention is currently limited.

## Abbreviations

BMI: Body mass index
BP: Blood pressure
CHD: Coronary heart disease
HbA1c: Glycated hemoglobin
HDL-C: High-density lipoprotein cholesterol
LDL-C: Low-density lipoprotein cholesterol
MCI: Mild cognitive impairment
MetS: Metabolic syndrome
TG: Triglycerides
TIA: Transient ischemic attack
